# Quantitative Histomorphometry of the Healthy Peritoneum

**DOI:** 10.1038/srep21344

**Published:** 2016-02-19

**Authors:** Betti Schaefer, Maria Bartosova, Stephan Macher-Goeppinger, Akos Ujszaszi, Markus Wallwiener, Joanne Nyarangi-Dix, Peter Sallay, Dorothea Burkhardt, Uwe Querfeld, Viktoria Pfeifle, Bernd Lahrmann, Vedat Schwenger, Elke Wühl, Stefan Holland-Cunz, Franz Schaefer, Claus P. Schmitt

**Affiliations:** 1 Center for Pediatric and Adolescent Medicine, University of Heidelberg, Germany; 2 Institute of Pathology, University of Heidelberg, Germany; 3 Institute of Pathophysiology, Semmelweis University, Budapest, Hungary; 4 Department of Obstetrics and Gynecology, University of Heidelberg, Germany; 5 Department of Urology, University of Heidelberg, Germany; 6 First Department of Pediatrics, Semmelweis University, Budapest, Hungary; 7 Department of Pediatric Nephrology, University of Charité, Berlin, Germany; 8 Division of Pediatric Surgery, University of Heidelberg, Germany; 9 Department of Pediatric Surgery, University Children´s Hospital Basel, Switzerland; 10 Bioquant, Hamamatsu Tissue Imaging and Analysis (TIGA) Center, Heidelberg, Germany; 11 Division of Nephrology, University of Heidelberg, Germany

## Abstract

The peritoneum plays an essential role in preventing abdominal frictions and adhesions and can be utilized as a dialysis membrane. Its physiological ultrastructure, however, has not yet been studied systematically. 106 standardized peritoneal and 69 omental specimens were obtained from 107 patients (0.1–60 years) undergoing surgery for disease not affecting the peritoneum for automated quantitative histomorphometry and immunohistochemistry. The mesothelial cell layer morphology and protein expression pattern is similar across all age groups. Infants below one year have a thinner submesothelium; inflammation, profibrotic activity and mesothelial cell translocation is largely absent in all age groups. Peritoneal blood capillaries, lymphatics and nerve fibers locate in three distinct submesothelial layers. Blood vessel density and endothelial surface area follow a U-shaped curve with highest values in infants below one year and lowest values in children aged 7–12 years. Lymphatic vessel density is much lower, and again highest in infants. Omental blood capillary density correlates with parietal peritoneal findings, whereas only few lymphatic vessels are present. The healthy peritoneum exhibits major thus far unknown particularities, pertaining to functionally relevant structures, and subject to substantial changes with age. The reference ranges established here provide a framework for future histomorphometric analyses and peritoneal transport modeling approaches.

The peritoneum is a delicate, continuous tissue layer that lines the peritoneal cavity^1^. As early as in 1897, Robinson defined its physiological functions of regulation of fluid for nutrition, facilitation of motion and prevention of friction^2^ and in the 20th century its suitability as a dialyzer membrane was established^3^,^4^. Since then, peritoneal dialysis (PD) has become the preferred mode of dialysis in children.

The functions of the peritoneum have been explored in numerous experimental and clinical studies and transperitoneal solute and water transport has also been modeled mathematically^5^. The mesothelial cell layer plays a key role in peritoneal homeostasis, host defense and prevention of local erosions and adhesions by secretion of cytokines, anticoagulants, surface active phospholipids, proteoglycans and lubricants^6^,^7^,^8^. The primary, rate-limiting barrier for solute and water transfer is formed by the submesothelial capillaries, which consist of endothelial cells linked by tight junctions and surrounded by a basement membrane^6^. Lymphatic capillaries reabsorb fluid, cells and macromolecules from the interstitial space into the circulation. The interstitial space is composed of extracellular matrix, i.e. bundles of collagens and mucopolysaccharides, and a limited number of cells such as fibroblasts, mononuclear cells, and nerve fibers. In case of excessive matrix deposition and fibrosis the interstitium can alter the resistance to fluid and solute transport^9^.

In contrast to the ample knowledge on the functions of the peritoneal tissue components, surprisingly little information is available about the morphological ultrastructure of the healthy peritoneum. Only rough descriptions of the peritoneal membrane have been provided based on samples from few healthy adults^10^,^11^ and children^12^. Precise knowledge of the anatomical make-up of the peritoneal membrane across the entire age range is crucial for advanced mathematical modeling of peritoneal transport functions, as well as for the interpretation of structural changes occurring during PD therapy. To meet this need, we obtained standardized peritoneal biopsies from a large cohort of healthy children and adults in a prospective multicenter study and performed automated quantitative histomorphometry as well as immunohistochemistry of key cells and proteins involved in peritoneal homeostasis and membrane transport function.

## Results

A total of 106 parietal and 69 visceral peritoneal samples were obtained in 107 patients. Biopsy sampling was well tolerated; no biopsy procedure related adverse events were reported. 70, 22 and 8% of the samples were obtained from the upper, middle and lower abdomenm, respectively. 76% of the samples were taken from the lateral wall and 24% from the ventral abdominal wall area. There was no systematic variation in the key histological parameters, i.e. mesothelial integrity and appearance, submesothelial thickness and microvessel density, in relation to the sampling site.

### Parietal peritoneum

#### Mesothelium

The mesothelium was positive for calretinine, podoplanin, Wilms tumor gene 1 (WT1), aquaporin 1 (AQP1), E-cadherin and cancer antigen 125 (CA125). The mesothelial cell layer was present in 84 of the 106 peritoneal biopsy samples, while in 22 (21%) the surface was denudated possibly due to preservation artifacts. The expression of mesothelial cell markers was constant across the age groups.

#### Submesothelium

**Figure 1:**
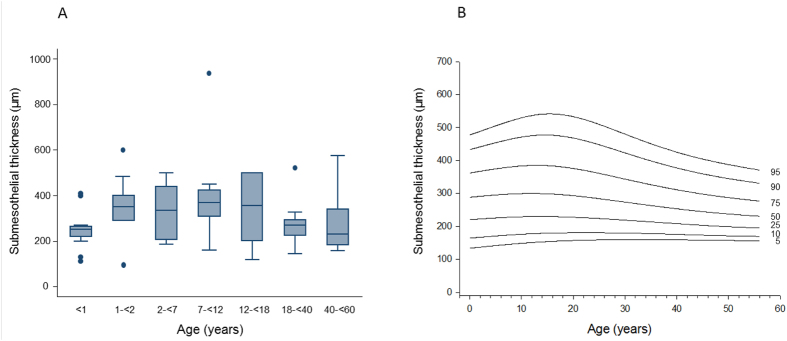
Age related thickness of the submesothelial peritoneum in healthy individuals given as (**A**) box plots and (**B**) percentile curves (p = 0.01).

Submesothelial thickness increased with age from infancy [median 230 (IQR 60 μm)] to mid childhood [402 (168) μm in 7–12 year old children] (p = 0.01) and was again lower in adults [173 (146) μm; p = 0.01; Fig. 1A]. Percentiles of peritoneal submesothelial thickness are given in Fig. 1B. A separate superficial submesothelial compact zone as previously described in adult PD patients^10^ could not be delineated. Submesothelial fat was present in 61% of the samples, adjacent muscle in 5%, fat and muscle in 17% and muscle fascia was seen in 17% of the samples. Body mass index (BMI) standard deviation score (SDS) was significantly higher in children with adjacent submesothelial fat [−0.06 ± 0.23 vs. −0.96 ± 0.4; p = 0.03].

**Figure 2:**
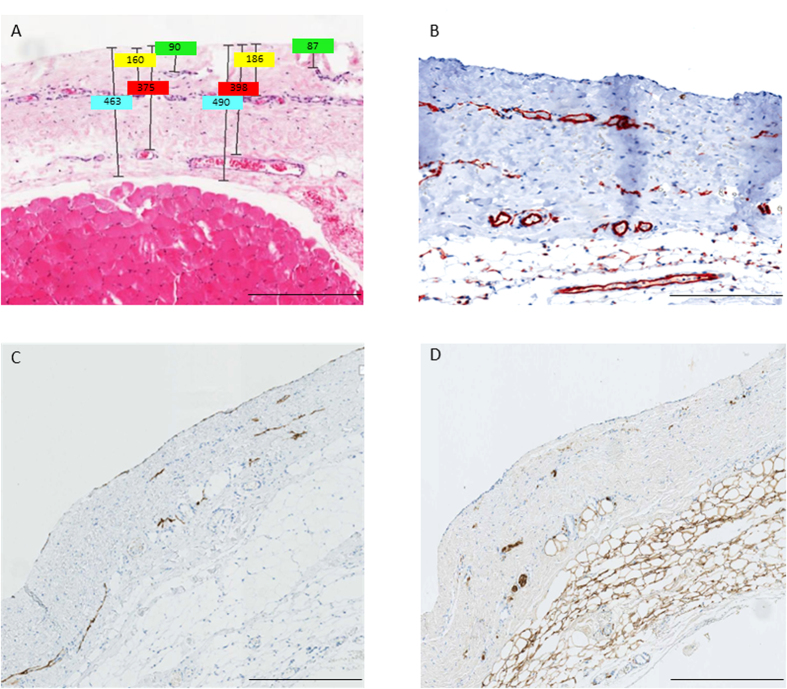
Hematoxylin-eosin (**A**) and CD31 (**B**) stainings of parietal peritoneum demonstrating the three layer structure (**A**,**B**) and co-localization of podoplanin positive lymphatic capillaries (**C**) and S100A4 positive nerve fibers (**D**). Scale bars: 400 μm. Illustrative examples of measurements of submesothelial thickness, and of the distance of the three vessel layers to the mesothelial surface are given in Fig. 2A.

Blood capillaries and lymphatic vessels of the peritoneal membrane are composed of a single layer of endothelial cells, expressing the endothelial cell marker CD31. The CD31 positive vessels were mostly located in three distinct submesothelial vessel layers, median 36 (IQR 20), 96 (65) and 192 (133) μm below the mesothelial cell monolayer and were accompanied by nerve fibers (Fig. 2). The distance of each vessel layer to the peritoneal surface was highly correlated with total submesothelial thickness (rho = 0.566, 0.599, 0.600; all p < 0.001).

**Figure 3:**
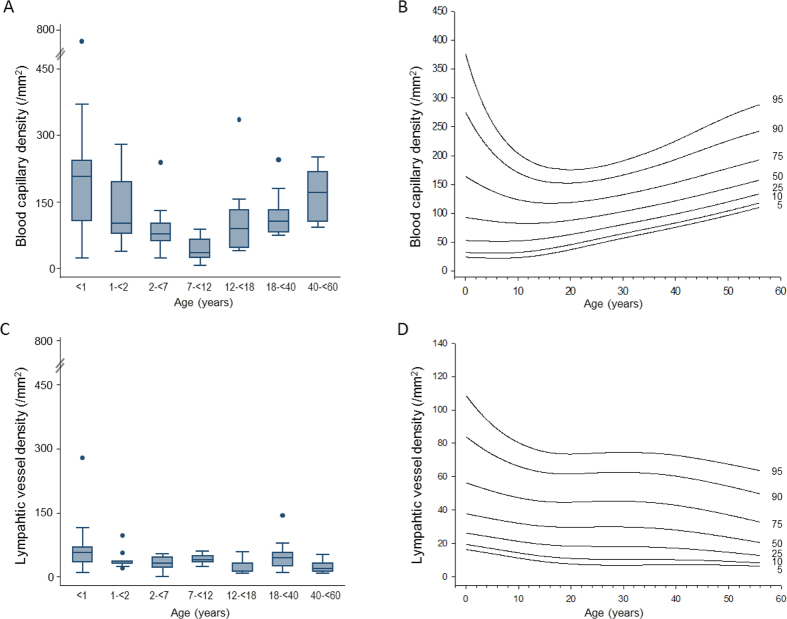
(**A**) Age dependent blood capillary density and (**C**) lymphatic capillary density and (**B,D**) respective percentile curves in healthy individuals (both p < 0.001).

**Figure 4:**
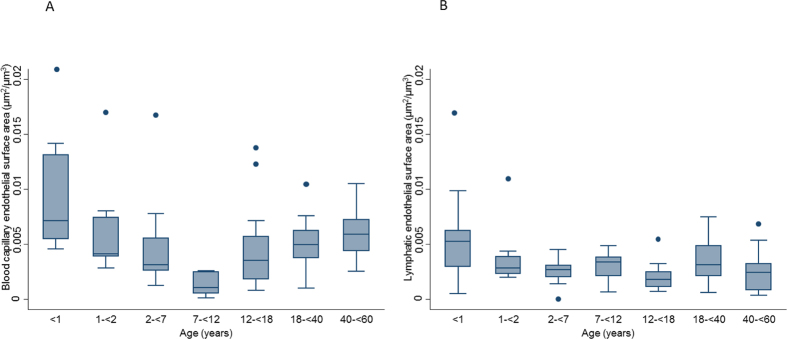
(**A**) Blood and (**B**) lymphatic endothelial surface area per parietal peritoneal volume over age (both p < 0.001).

Total peritoneal microvessel density strongly depended on age and was highest in children below 1 year (281/mm^2^, IQR 94), markedly lower in children below 2, 7, 12 and 18 years [126 (56); 102 (73); 72 (34); and 97(49)] and again significantly higher in adults [152 (47); 176 (111); p < 0.001]. Likewise, blood capillary density followed a U-shaped curve with highest values in infants, lowest in children and higher values again in adults (p < 0.001; Fig. 3). The morphology of blood capillaries was similar in all age groups except for endothelial thickness, which showed an inverse U-shaped association with age (p = 0.01; Table 1). The relative endothelial area of blood capillaries per peritoneal wall area available for solute and water transport was 5.8 (2.0), 1.9 (1.2), 1.4 (1.4), 1.9 (1.4), 2.3 (1.9), 2.0 (1.6), and 2.2 (2.0) % at <1, 2, 7, 12, 18, 40 and 60 years of age (p = 0.01). Likewise, blood capillary endothelial surface area per volume of peritoneal membrane was threefold higher in the first year of life (p < 0.001; Fig. 4A).

The density of the lymphatic vessels was markedly lower than that of blood vessels at all ages, but again 70% higher in infants (p < 0.001; Table 2; Fig. 3). Lymphatic endothelial area relative to peritoneal wall area was 1.4 (0.7), 0.5 (0.2), 0.5 (0.3), 0.6 (0.4), 0.3 (0.8), 0.7 (0.8) and 0.7 (0.7) % at <1, 2, 7, 12, 18, 40 and 60 years of age (p = 0.01). Likewise, lymphatic endothelial surface area per volume of peritoneal membrane was threefold higher in infants below one year (p < 0.001; Fig. 4B). Reference percentile curves of peritoneal blood and lymphatic vessel density are given in Fig. 3.

None of the peritoneal capillaries showed any signs of vasculopathy, i.e. of luminal narrowing or obliteration. Sparse alpha smooth muscle actin (ASMA) positive, activated fibroblasts were observed in 24% of the parietal peritoneal samples across all age groups (p = 0.5). In one 15 year old child, a small cluster of activated fibroblasts was detected; Acid Fuchsin Orange G (AFOG) staining ruled out fibrin depositions. Transforming growth factor ß (TGF-ß) inducible phosphorylation of SMAD2/3 was largely absent; only traces of pSMAD2/3 could be stained in the submesothelium in 10% of the peritoneal samples. Few, isolated inflammatory cells were present, i.e. CD45 positive lymphocytes in 12% and CD68 positive macrophages in 10% of the cases. Independent of age, tryptase positive mast cells were present in all samples at low levels (12.9 ± 1.1 cells/mm^2^). No cytokeratin positive mesothelial cells were present in the submesothelium; double staining with fibroblast specific protein 1 (FSP-1) was negative, indicating that epithelial-to-mesenchymal transition (EMT) did not occur. Calcifications were not detected. AQP-1 and vascular endothelial growth factor A (VEGF-A) are expressed in mesothelial and endothelial cells. The AQP-1 protein abundance of the submesothelium was correlated with microvessel density (r = 0.534; p = 0.01), whereas VEGF-A abundance was low and unrelated to vessel density.

### Omental peritoneum

The omentum was covered by a podoplanin positive mesothelial cell layer with similar appearance of flat and adjacent cells as the parietal peritoneum. The submesothelial area contained adipose tissue, capillaries, and isolated bundles of large vessels. The density of CD31 positive capillaries was measured in submesothelial areas corresponding to the thickness of the respective parietal peritoneal sample. Similar to the parietal peritoneum, omental vessel density was significantly higher in children younger than 2 years as compared to older age groups. Omental vessel density was 434 (200), 151 (312), 18 (50), 24 (133), 83 (6), 44 (118) and 36 (110)/mm^2^ below 1, 2, 7, 12, 18, 40 and 60 years of age (p = 0.01). In contrast, lymphatic capillaries were few with only 20 (32), 6 (7), 2 (5), 3 (3), 5 (10), 7 (7), and 4 (13)/mm^2^ below 1, 2, 7, 12, 18, 40 and 60 years of age (p = 0.2). Parietal peritoneal and omental vessel density were correlated (r = 0.391; p = 0.03) and the morphological characteristics of the capillaries were comparable.

Elastica van Gieson (EVG) staining and quantitative analysis was performed on three omental arterioles in each of 48 patients. Endothelial thickness was 1.04 (0.33), 1.2 (0.23), 1.36 (0.56), 1.28 (0.34), 1.29 (0.4) and 1.55 (0.34) μm in individuals <1, 2, 7, 12, 18 and 40 years (p = 0.07). Arteriolar endothelial thickness was closely correlated with the thickness of the tunica media (rho = 0.65; p < 0.001).

Total blood and lymphatic microvessel density of the omental and parietal peritoneum showed similar associations to body surface area, body length and weight, but were independent of gender and age standardized BMI scores.

## Discussion

This is the first comprehensive analysis of the morphology of the healthy peritoneal membrane encompassing the pediatric and adult age range, providing an important contribution to the understanding of the mechanics of peritoneal dialysis across ages.

**Figure 5:**
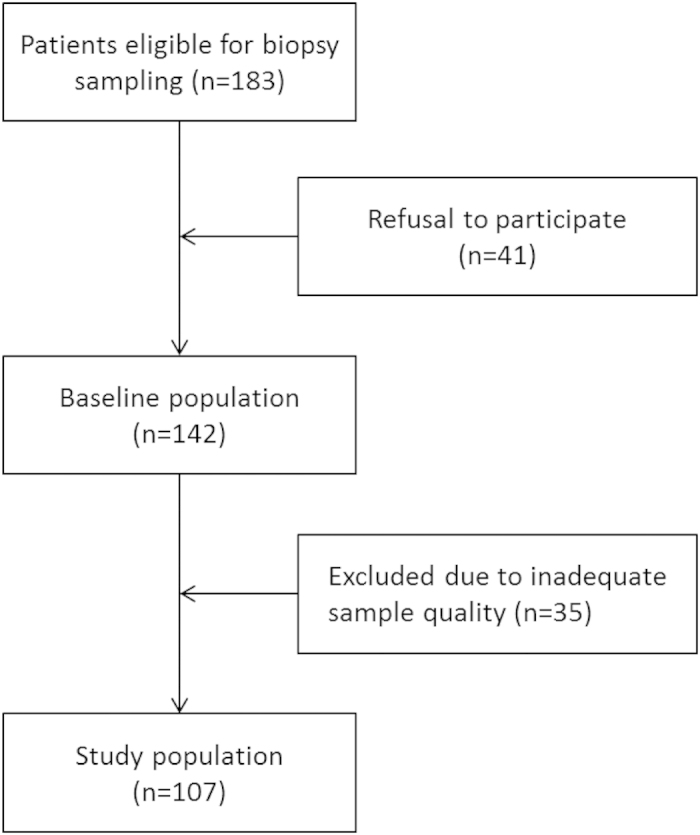
Patient selection chart.

Utmost care was taken to optimize methodological quality in this prospective multicenter study. Local collection and pre-analytical processing of samples was performed according to a standardized protocol, histopathological processing and analysis were performed centrally. An automated quantitative image analysis system was used to minimize observer bias. One quarter of the parietal tissue samples were excluded from analysis (Fig. 5) mainly since these were too small and thin, i.e. not comprising structures adjacent to the peritoneum and/or not fixed appropriately for preservation of the delicate structures. Among the remaining samples 20% still had major defects in the mesothelial monolayer, which presumably developed during sample processing.

We observed a consistent age related change of peritoneal capillary density, which was more than twice as high in young infants as in older children. The latter phenomenon may in part be explained by the rapid increase in body size during infancy. A constant number of capillaries contained in an increasing tissue volume will predictably lead to a decrease of vessel density. A similar phenomenon has been observed for the number of glomeruli retrieved with a given cone size by kidney biopsy in children of different ages^13^.

The solute and water transport capacity of the peritoneum depends on its blood capillary density and perfusion^9^. According to computational studies it moreover depends on lymphatic reabsorption rates, increasing with interstitial tissue pressure, and on the spatial vessel distribution within the submesothelium. The upper 2–3 mm of the peritoneum participate in the exchange of fluid and solutes with 90% of the concentration changes occurring within the first 400 μm^14^,^15^. Biopsy studies in adult PD patients demonstrated a significant increase in peritoneal vessel density and submesothelial thickness with time on PD, however, did not relate these findings to peritoneal transport function and UF capacity^10^,^16^. In a small study comprising 35 PD patients vessel density did not correlate with transporter status^17^. The higher blood vessel density of young infants is in line with the increased small solute mass transport area coefficients found in this age group^18^. The lower ultrafiltration capacity - due to higher glucose resorption capacity - in infants is reflected clinically by the need for frequent short-dwell exchanges^19^. Capillary density was lowest in children of school age, and increased again in adults. We speculate that this finding might reflect a certain degree of angiogenesis related to vascular ageing. A possible age dependency of peritoneal transport function in adults has not been studied systematically. Our findings would indicate increasing solute transport and decreasing ultrafiltration capacity with age in adults.

A further striking finding is the higher density of lymphatic vessels in young children. Long dwell times with dissipation of the osmotic gradient and high intraperitoneal pressure result in reabsorption of ultrafiltrate and solvents into the submesothelial interstitium. While water is reabsorbed along the Starling forces into the blood capillaries, larger molecules are mainly reabsorbed via lymphatic vessels. Based on dextran uptake, the lymphatic absorption rate has been estimated to be 1.5–2 ml/min/1.73 m^2^ in children aged 1–18 years, with 70% higher rates measured in infants younger than two years of age^20^,^21^. Lower ultrafiltration rates are achieved with icodextrin fluid in infants than in older children, possibly due to higher icodextrin absorption^22^. The higher density of lymphatic vessels in young infants found in this study provides a morphological correlate to these functional observations.

Another novel finding of this study is the predominant alignment of blood and lymphatic vessels together with nerve fibers in three distinct layers at distances closely related to the total submesothelial thickness. This finding, together with the observed distinct age related changes in capillary density and submesothelial thickness, informs future approaches to peritoneal transport modeling^5^. Also, it will be interesting to explore how the three layer microvascular structure will transform when PD induced angiogenesis occurs.

Peritoneal inflammation, angiogenesis and fibrosis inevitably develop during extended PD^10^ and limit PD utilization. Our study provides important reference information about the background level of inflammatory activity in the healthy peritoneum. In the absence of PD and local or systemic inflammatory disease, the parietal peritoneum contains only few isolated active fibroblasts and inflammatory cells and scarce fibrin deposits with no detectable epithelial-to-mesenchymal transition of mesothelial cells. Of note, the concept of mesothelial cells migrating into the submesothelium and transforming into a myofibroblast phenotype has been challenged by one recent paper^23^. VEGF and TGF-ß inducible SMAD2/3 phosphorylation were hardly detectable, suggesting that these cytokines, which are key mediators of the transformation of the peritoneal membrane in patients undergoing PD, play a negligible role in the healthy peritoneum^24^. The mesothelial cell monolayer, which plays an essential role in maintaining local peritoneal homeostasis and preservation of peritoneal surface integrity, exhibits no age dependent particularities. Morphology and specific protein expression pattern are similar throughout all age groups, even in very young infants where some immaturity might have been expected.

Altogether these findings, including the reference ranges established for peritoneal thickness and blood and lymphatic vascularization from age 0 to 60 years, will allow for standardized analysis and interpretation of future histopathological studies in patients undergoing peritoneal dialysis.

The omentum plays an important role in the peritoneal defense machinery and, at least in experimental models, essentially contributes to peritoneal tissue remodeling during PD^25^. The relative contribution of the omental peritoneum to PD transport function in humans is unknown and probably less than that of the parietal peritoneum, given the limited omental surface area. The tissue fine structure differs from the parietal peritoneum, with the omentum mainly consisting of fat tissue and blood capillaries but very few lymphatic vessels. On the other hand, we found omental and parietal blood vessel density to be significantly correlated. Hence, omental tissue harvested during abdominal surgery may be informative of a patient’s microvascular status. Also, similar histopathological changes have been observed in omental and parietal peritoneum in disease conditions, such as fibrosis and lymphangiogenesis in patients with encapsulating peritoneal sclerosis^26^,^27^.

In conclusion, our systematic analysis of the peritoneal fine structure revealed several hitherto unknown features such as the configuration of the vessels and nerves within three distinct layers and marked age dependent changes in peritoneal blood and lymphatic vascularization, with highest values in infants and lowest in school children. Our findings and derived reference ranges provide a framework for clinical studies as well as for future approaches to model the mechanics of peritoneal mass transport.

## Methods

### Patients

Between February 2011 and March 2015 183 patients were screened for inclusion into the study in Heidelberg, Budapest and Berlin. Patients with a BMI of >35 kg/m^2^ were excluded. 41 refused participation and 35 patients had to be excluded for inadequate sample quality (Fig. 5). From the remaining 107 patients (age 1 month - 60 years) 106 parietal and 69 omental biopsy samples were obtained. The patients were divided into seven age groups: <1, 1-<2, 2-<7, 7-<12, 12- <18 and 18–40, 41–60 years and included 14, 10, 15, 15, 18, 21 and 14 patients, respectively. Blood was sampled the day prior to surgery and a whole blood count, C reactive protein, serum creatinine and electrolytes determined by standard laboratory methods. Hemoglobin levels were physiologically lower in infants than in every other age group [11.5 ± 0.5 vs. 12.9 ± 0.2 g/dl; p = 0.022]. Likewise leukocytes [10.7 (3.1) vs. 7.7 (3.6) G/l; p = 0.003] and thrombocytes [452 (258) vs. 296 (129) G/l; p = 0.002] were higher in infants, but within the normal range. CRP levels were consistently low [2(3), 2(0), 2(0), 2(0), 2(0), 2(4), 3(2); p = 0.752], and indicate a non-inflammatory status. Serum creatinine levels show a physiological increase with age from 19.9 (5.3) mmol/l in infants to 60.1 (16.8) mmol/l in adults, serum calcium was higher in infants (2.47 ± 0.07 mmol/l) as in older patients (2.34 ± 0.02 mmol/l, p = 0.007). Serum sodium was 140 ± 0.3 mmol/l; p = 0.879, serum potassium was 4.2 ± 0.1; p = 0.098 and were not different between age groups. None of the patients had diseases affecting the peritoneum, clinical or biochemical signs of systemic inflammation or renal failure. Indications for the surgery were gastrointestinal in 46 of the patients [laparoscopic fundoplication (n = 33), cholecystectomy (5), pyloromyotomy (2), other (6)], urological in 32 patients [kidney or urinary tract malformation/obstruction (15), benign kidney cysts/tumor (6), living kidney donation (8) other (3)], gynecological in 15 patients [myomectomy (7), ovarial cyst removal (6), salpingectomy (2)], splenectomy was performed in 4 patients, explorative laparotomy in 6, others in 4 cases.

The Ethical Committee of the Medical Faculty at the University of Heidelberg, at Charité Berlin and at Semmelweis University Budapest approved the study. Written informed consent was obtained from parents, and from patients as appropriate. The study was performed according to the Declaration of Helsinki and registered at www.clinicaltrials.gov (NCT01893710).

### Biopsy Sampling

Samples of the parietal peritoneum were obtained in a standardized manner as previously described^10^. Briefly, the peritoneum was exposed and a suture loop was inserted through the part of peritoneum to be sampled. A loose knot was tied; the peritoneum was lifted and, according to age, a 0.4–1 cm × 0.4–1 cm and 2–4 mm deep tissue fragment was excised. Immediately after excision, half of the peritoneal sample was fixed with needles on cork plate with the mesothelium uppermost and placed in 4% formalin, half of the sample immediately deep frozen in liquid nitrogen. Omental specimens were stored in formalin and deep frozen in liquid nitrogen, respectively.

### Histomorphometry

Each peritoneal sample underwent Iight microscopy. For assessment of the mesothelial cell layer integrity and age specific protein expression pattern, the mesothelial cell layer was stained for calretinin, podoplanin (D2–40), WT1 and CA125. All slides were scanned at an original magnification of 40x using a whole slide imaging system (ScanScope CS, Aperio^®^, Vista, California). The submesothelial zone of the parietal peritoneum was defined in hematoxylin-eosin (HE) stained sections as the zone underneath the mesothelial cell monolayer reaching down to the muscle, muscle fascia, or submesothelial fat cell layer, as present. At least five measurements were performed. Submesothelial capillary and lymphatic vessel density was measured in CD31, and lymphatic vessel density in podoplanin stained sections. Image analysis including quantification of vessels was performed using the Aperio^®^ image analysis toolbox. Three investigators (SGM, BS, MB) independently evaluated the samples.

### Immunohistochemistry

Immunohistochemistry was performed on formalin fixed tissue sections according to standard methods. Briefly, dewaxed and rehydrated tissue sections were incubated in 3% hydrogen peroxide to block endogenous peroxidases. The heat-induced antigen retrieval was performed in a microwave oven, using the 0.01 or 0.005 M citrate buffer, pH 6. The primary antibodies were applied for 1 hour or overnight. Incubation with biotinylated secondary reagents for 30 min was followed by the avidin-biotin complex (ABC) ABC reagent (both from Vector Laboratories, California, USA). 3′3′Diaminobenzidine (DAB) and Sirius Red (Sigma, Taufkirchen, Germany) were used for detection. Universal blocking reagent in phosphate buffered saline (PBS) (1:100) without primary antibody was used for negative control. Cell nuclei were counterstained with HE. HE, AFOG and EVG stainings were performed according to standard protocols.

Monoclonal antibodies against cluster of differentiation CD31, CD45, CD68, CA125 and D2–40 (podoplanin) and Anti-Mast Cell Tryptase Clone AA1 were purchased from Dako Cytomation (Glostrup, Denmark). The D2–40 antibody does not cross react with blood endothelia. Antibodies against Pan-Cytokeratin were obtained from Sigma Aldrich (St. Louis, Missouri, USA), against WT1 and E-cadherin from Cell Marque (Cell Marque Corporation, Rocklin, California, USA). The monoclonal AQP1 antibody purchased from Santa Cruz Biotechnology (Santa Cruz, California, USA) has no cross reactivity with other AQPs. The polyclonal antibody against pSMAD2/3 was purchased from Santa Cruz Biotechnology (Santa Cruz, California, USA), against calretinine from Cell Marque (Rocklin, California, USA), against neuronal marker calcium-binding protein A4 (S100A4) and FSP1 from Dako Cytomation (Glostrup, Denmark), and against VEGF-A from Abcam (Cambridge, UK).

### Automated quantitative analyses

**Figure 6:**
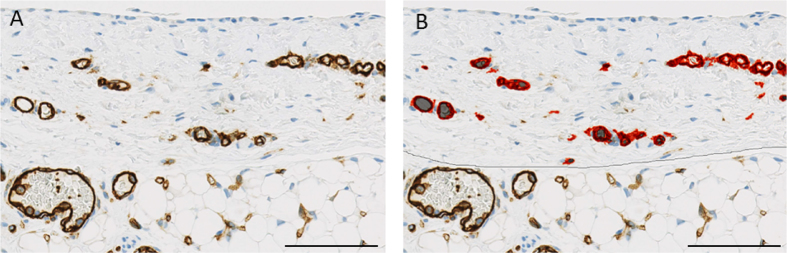
Illustration of a CD31 stained peritoneal tissue section (**A**) analyzed by Aperio, marking up endothelial area in red (**B**). Scale bar 100 μm.

Automated quantitative analyses were performed using the Aperio Image Analysis Software (Aperio® Technologies, Inc., Vista, California, USA) and viewed by Image Scope version 11 (v11.2.0.780). Microvessel analyses were performed using microvessel algorithm version 1 after color calibration for CD31 (staining both blood and lymphatic vessels) and podoplanin (staining lymphatic vessels and mesothelial cells). Total area analyzed per section was 1.44 (0.74–2.70) mm^2^. Findings were reconfirmed by visual counting. Vessels were defined as CD31 positive endothelial cells lining the inner vessel wall and typically with a lumen (Fig. 6). Microvessel density was defined as number of vessels per unit of analysis area. Blood vessel density was calculated from intraindividual differences in CD31 and podoplanin positive capillaries. Capillary vessel area was defined as the sum of endothelial area plus the lumen area and capillary wall by the endothelial layer thickness. Collapsed vessels, i.e. vessels without a distinct lumen, were excluded from analysis of vessel area, perimeter and endothelial thickness. The endothelial area is the sum of all endothelial cells without the lumen area. The capillary endothelial surface area relative to peritoneal volume was defined as the endoluminal perimeter of CD31 stained endothelium *section thickness *number of vessels divided by the analyzed peritoneal area *section thickness (μm^2^/μm^3^).

Immunohistochemical stainings were evaluated using the Aperio Positive Pixel Count Algorithm (version 9) for quantification of the amount of positive pixels per scanned virtual slide. Intensity ranges for weak, medium and strong signals and negative pixels were validated for each specific staining. Positivity was calculated as total number of positive pixels divided by total number of pixels.

### Statistics

Descriptive data were summarized using proportions, means (standard error of mean, SEM) or medians (interquartile range, IQR) as appropriate. Normal distribution was assessed graphically and by Shapiro-Wilk test. For continuous variables Pearson’s or Spearman correlations were used based on the data distribution. To compare data across groups ANOVA or Kruskal-Wallis test and for describing differences in proportions, chi^2^ or Fischer’s exact-test were used as applicable. In all statistics, two-sided tests were used and the results were considered statistically significant if p value was <0.05. The LMS method was applied as published previously^28^ for calculation of percentile curves accounting for non-linearity and skewed distributions of the reference data set.

## Additional Information

**How to cite this article**: Schaefer, B. *et al*. Quantitative Histomorphometry of the Healthy Peritoneum. *Sci. Rep*. **6**, 21344; doi: 10.1038/srep21344 (2016).

## Figures and Tables

**Table 1:** Peritoneal blood capillary density and morphology (median and IQR).

	<1 years	1-<2 years	2-<7 years	7-<12 years	12-<18 years	18-<40 years	40-<65 years	P value
Blood capillary density (/mm^2^)	223 (106)	89 (43)	76 (32)	35 (41)	89 (85)	106 (51)	172 (113)	<0.001
Area per vessel (μm^2^)	57 (12)	51 (38)	88 (61)	64 (107)	68 (39)	55 (29)	48 (22)	0.4
Vessel perimeter (μm)	43 (10)	35 (22)	50 (30)	30 (42)	43 (17)	44 (13)	42 (16)	0.6
Endothelial area per vessel (μm^2^)	53 (12)	40 (44)	89 (68)	63 (97)	68 (36)	54 (30)	52 (20)	0.3
Endothelial thickness (μm)	1.29 (0.32)	1.12 (0.58)	1.45 (0.52)	1.95 (1.28)	1.48 (0.42)	1.21 (0.42)	1.17 (0.25)	0.01

**Table 2:** Peritoneal lymphatic vessel density and morphology (median and IQR).

	<1 years	1-<2 years	2-<7 years	7-<12 years	12-<18 years	18-<40 years	40-<65 years	P value
Lymphatic vessel density (/mm^2^)	58 (35)	37 (6)	33 (23)	39 (14)	12 (19)	44 (32)	19 (20)	<0.001
Area per vessel (μm^2^)	112 (53)	87 (72)	71 (94)	75 (47)	106 (80)	83 (63)	177 (314)	0.4
Vessel perimeter (μm)	84 (37)	80 (30)	69 (51)	78 (29)	76 (49)	79 (44)	102 (120)	0.8
Endothelial area per vessel (μm^2^)	106 (48)	80 (75)	66 (90)	75 (44)	99 (90)	83 (63)	156 (240)	0.5
Endothelial thickness (μm)	1.15 (0.28)	0.99 (0.21)	0.94 (0.23)	1.00 (0.19)	1.17 (0.67)	1.07 (0.27)	1.32 (0.45)	0.06

Number of patients per group analyzed for blood and lymphatic vessel density are 14, 10, 15, 15, 18, 21, 14, for both tables, respectively.
